# Transparent Hybrid Opals with Unexpected Strong Resonance‐Enhanced Photothermal Energy Conversion

**DOI:** 10.1002/adma.202004732

**Published:** 2020-11-30

**Authors:** Yu Cang, Jaejun Lee, Zuyuan Wang, Jiajun Yan, Krzysztof Matyjaszewski, Michael R. Bockstaller, George Fytas

**Affiliations:** ^1^ School of Aerospace Engineering and Applied Mechanics Tongji University 100 Zhangwu Road Shanghai 200092 China; ^2^ Max Planck Institute for Polymer Research Ackermannweg 10 Mainz 55128 Germany; ^3^ Department of Materials Science and Engineering Carnegie Mellon University 5000 Forbes Avenue Pittsburgh PA 15213 USA; ^4^ Chemistry Department Carnegie Mellon University 4400 Fifth Ave. Pittsburgh PA 15213 USA

**Keywords:** absorption enhancement, particle brush systems, photothermal energy conversion, slow photon

## Abstract

Photothermal energy conversion is of fundamental importance to applications ranging from drug delivery to microfluidics and from ablation to fabrication. It typically originates from absorptive processes in materials that—when coupled with non‐radiative dissipative processes—allow the conversion of radiative energy into heat. Microstructure design provides versatile strategies for controlling light–matter interactions. In particular, the deliberate engineering of the band structure in photonic materials is known to be an effective approach to amplify absorption in materials. However, photonic amplification is generally tied to high optical contrast materials which limit the applicability of the concept to metamaterials such as microfabricated metal–air hybrids. This contribution describes the first observation of pronounced amplification of absorption in low contrast opals formed by the self‐assembly of polymer‐tethered particles. The dependence of the amplification factor on the length scale and degree of order of materials as well as the angle of incidence reveal that it is related to the slow photon effect. A remarkable amplification factor of 16 is shown to facilitate the rapid “melting” of opal films even in the absence of “visible” absorption. The results point to novel opportunities for tailoring light–matter interactions in hybrid materials that can benefit the manipulation and fabrication of functional materials.

## Graphical abstract

Optical absorption, being absent in the pure polymer matrix and that with ordered particles, is remarkably amplified in self‐assembled polymer‐tethered (brush) particles comprising low‐contrast constituents. The amplification is shown to be caused by “photon slowdown” and to induce rapid “melting” of materials even in the absence of “visible” absorption.

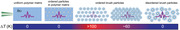

The manipulation of light–matter interactions through microstructure design have emerged as an important concept in the context of light‐enabled technologies.^[^ ^1^ ^]^ Owing to the advancements of fabrication technologies, tailoring of the structure at micro‐/nanoscale allows precisely and efficiently manipulating the flow of light. This has been demonstrated to play a critical role in the high performance of light‐related devices including photocatalysis, photovoltaics, photodetectors, and light‐emitting diodes.^[^ ^2^ ^]^ Light management strategies based on micro‐/nanostructure include scattering enhancements, antireflection, resonant, photonic crystals, and plasmonics. In such structures, strong light trapping can greatly enhance the absorption in a wide range of wavelengths.^[^ ^3^ ^]^

“Light‐harvesting structures” based on photonic crystals have been applied in photovoltaic devices^[^ ^4^ ^]^ complementing alternative methods, such as the luminescent downshifting technique. In the latter strongly absorbed short‐wavelength photons are down‐converted into a more efficient visible range, enhancing the power conversion efficiency.^[^ ^5^ ^]^ The purpose of the photonic crystal approach is to increase the probability of photon absorption in semiconductor active layers by prolonging the “residence time” of light, consequently enhancing the energy conversion efficiency.^[^ ^6^ ^]^ The increase of the “residence time” of light is facilitated by harnessing a particular feature of photonic crystals known as the “slowdown” of photons with frequencies near the edge of the respective photonic bandgap.^[^ ^7^ ^]^ This “slowdown” can be thought to originate from the vanishing slope of the photon dispersion curves dω/d*k* (with ω and *k* denoting the light frequency and the magnitude of the light wave vector, respectively) near the edges of a bandgap.^[^ ^8^ ^]^ Since the curvature is inversely related to the lifetime of the Bloch mode that characterizes the propagation of light in a photonic crystal, the photons in the edge regions of a gap can be thought to travel with vanishing (group) velocities (aka “slow photon”).^[^ ^8^, ^9^ ^]^ The slowdown increases the interaction of light with the material and the possibility for energy transfer. This has been used in a variety of contexts such as solar energy^[^ ^7^, ^8^, ^10^ ^]^ and photocatalysis.^[^ ^11^ ^]^

The “slow photon” effect has thus expanded the scope of “materials for photothermal energy conversion” beyond the established domain of strongly absorbing materials such as 2D‐black phosphorous and bismuth selenide, which have attracted interest, for example, for biomedical applications.^[^ ^12^ ^]^ The amplification of the optical absorption enabled by the “slow photon” effect not only allows the application of less absorbing materials (which is beneficial depending on the application) but also reduces the amount of material necessary to achieve the desired level of photothermal energy conversion.

Here we report an unexpected resonance‐enhanced photothermal energy transfer process occurring in soft opals based on self‐assembled brush‐grafted particles (brush: polystyrene (PS), particle: silica (SiO_2_)) with a low optical contrast. This enhancement bears similarity to photoelectric or photochemical processes facilitated by the photonic slowdown described above. We classify the observed effect as “unexpected” because it occurs in self‐assembled structures of low optical contrast, whereas previous reports on photothermal energy conversion in photonic crystals were limited to microfabricated metal‐in‐air structures with “perfect” regularity and large optical contrast.^[^ ^11^, ^13^ ^]^ To probe the temperature variation, we utilize Brillouin light spectroscopy (BLS), a sensitive photoacoustic technique which non‐destructively evaluates the elastic modulus of the opals during irradiation. We first calibrate a sound velocity versus temperature relation by conducting BLS measurements under isothermal conditions with a low laser input power. For opals with the band edge approximately coincident with the wavelength of the probing laser (λ = 532 nm), rapid softening of the polymer matrix is observed. In contrast, no softening occurs under non‐resonant conditions that are realized by changing either the lattice period of the opals or the incident angle of the probing laser. Interestingly, the energy absorption under resonant conditions suggests an energy absorption coefficient of about 200 m^−1^, although no detectable light absorption occurs in the pristine particle or brush materials. This indicates a significant amplification of radiation energy conversion in the appropriate resonant conditions that could find applications, for example, as means for light‐driven forming or processing of polymer‐based materials.

The material systems, PS‐grafted SiO_2_, were synthesized using surface‐initiated atom transfer radical polymerization (SI‐ATRP) as described previously.^[^ ^14^ ^]^ Various particle sizes and grafting conditions were evaluated to assess the impact of lattice periodicity and degree of order. Photonic (iridescent) behavior was observed only for opal systems assembled from brush particles with a radius of the silica core *R* = 57 nm and submicron spacing. By varying the grafting density and degree of polymerization, we prepared soft opals with interparticle (core‐to‐core) distances ranging from 34 to 318 nm. Smaller silica particles (*R* = 7 nm) were evaluated as reference materials because of their larger intrinsic absorption cross‐section that is a consequence of the increased number of chain‐end functionalities (see discussion below). Similarly, larger SiO_2_ particles (*R* = 110 nm) were evaluated because of their larger optical scattering cross‐section, to confirm that the observed resonance‐enhanced photothermal energy conversion was not related to multiple scattering in the film; DP955 and DP955/DP214 (**Table** **1**) with the largest core size display stronger multiple scattering than all other systems. The characteristics of the particle‐brush systems studied in this work are listed in Table 1 (sample ID: DP*N*, where *N* represents the degree of polymerization of the PS brush) and Table S1, Supporting Information, which summarizes the elastic properties and thickness of all film samples. We note that the soft opals of 57 nm silica particles exhibited a grafting density in the range of σ = 0.27–0.53 nm^−2^. At these rather high grafting densities, the tethered chains are expected to adopt a stretched conformation in the vicinity of the particle surface.^[^ ^15^ ^]^ All nanocomposite films are assumed to have similar glass transition temperatures.^[^ ^16^ ^]^ Soft opal films with thicknesses in the range of 100–150 µm were fabricated by drop‐casting from 5% solutions in toluene and subsequent thermal annealing for 24 h at *T* = 120 °C. Previous work revealed that, under these conditions, face‐centered‐cubic (fcc) opal structures are formed.^[^ ^17^ ^]^ The peak wavelength of reflection of DP530 was found at λ_ref_ = 365 nm (Figure S1, Supporting Information), whereas films formed by DP1300 and DP2480 were not found to exhibit iridescence in the visible range. This was consistent with the larger periods of DP1300 (*d =* 246 nm) and DP2480 (*d* = 275 nm).

**Table 1:** Characteristics of the PS‐grafted SiO_2_ particle brush systems in this work

Sample ID	Grafting density σ [nm^−2^]	Degree of polymerization *N*	Radius of silica core^a)^ *R* [nm]	Core‐to‐core distance^a)^ *d* [nm]
DP600	0.04	600	7	34
DP530	0.27	530	57	163
DP1300	0.53	1300	57	246
DP2480	0.39	2480	57	275
DP214	0.92	214	110	260
DP955	0.43	955	110	318

^a)^ Determined from transmission electron microscopy images shown in Figures 1 and 3.

First, films were irradiated with a Nd:YAG diode laser (λ = 532 nm) at a power *P* = 10–150 mW for different durations. Concurrent BLS measurement was performed to determine the sound velocity, *c*(*P*), in the material as a sensitive in situ probe of the temperature in the illuminated volume. Comparison with the *c*(*T*) calibration data that was obtained previously for the soft opal systems in the present study (but at a sufficiently low laser power *P* = 5 mW to avoid laser‐heating‐induced temperature changes during the experiment) enabled the in situ determination of the local steady‐state temperature within the illuminated region of the films. Independent infrared (IR) imaging measurements of the samples were performed to validate the measured *T* changes. It should be noted that the possibilities of “degradation”, such as (photo) oxidation and physical aging, are negligible in the particle‐brush systems. Physical properties and microstructures were retained during the entire course of the study (approx. three years). The long‐term stability of material systems was further confirmed by the reproducibility of results over the entire experimental period. Finite element method (FEM) simulations were performed to deduce the effective absorption coefficient of each film by matching the experimental temperature change during illumination.

**Figure** **1** depicts representative BLS results for three samples with different lattice periods utilizing the transmission scattering geometry illustrated in Figure 1a. At this geometry, the corresponding phonon wave vector, **q**, was parallel to the {111} plane of the fcc packed film (i.e., parallel to the film surface). The magnitude of **q** is given as *q* = (4π/λ)sinα, where λ = 532 nm and the scattering angle 2α was in the range of 30° to 120°. Figure 1b,c depict representative BLS results for DP1300 with *d* = 246 nm as the input power *P* increased from 10 to 150 mW. The polarized BLS spectra (Figure 1b) recorded at *q* = 0.00808 nm^−1^ display a single peak at a frequency *f*, which red‐shifts as *P* increases. The strong decrease in the corresponding longitudinal sound velocity, *c* = 2*πf*/*q*, with the input power as shown in Figure 1c and Figure S2, Supporting Information, indicates irradiation‐induced softening of the opal films. As expected, the sound velocity in films of pristine PS and SiO_2_ is robust with respect to the variation of the radiation power (Figure S3, Supporting Information). Given that all measurements were performed under ambient conditions, the changes in the sound velocity could be attributed to the variation of temperature and/or composition.^[^ ^16^ ^]^ However, the robust phonon dispersion relation of DP1300 upon irradiation (Figure S2, Supporting Information) suggests no structural change before and after glass transition, thus ruling out the possibility of local composition change as the cause for the observed variation in the sound velocity.

**Figure 1:**
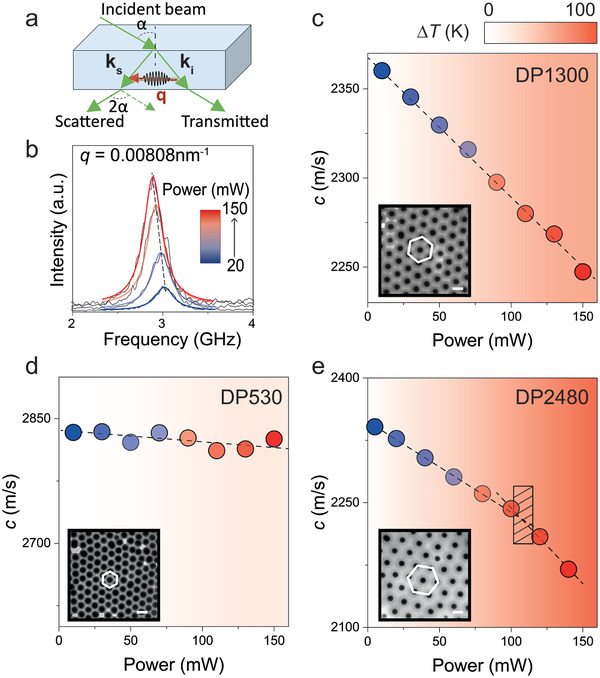
Control of the optical absorption in the visible range by sub‐micrometer assembly. a) Schematic of the transmission scattering geometry employed in the BLS experiments. The phonon wave vector **q** = **k**_s_ − **k**_i_ is in the plane of the film, where **k**_i_ and **k**_s_ are wave vectors of the incident and scattered light, respectively. The scattering angle (between the incident and scattered light) is twice the incident angle α. b) Evolution of the power‐dependent BLS spectra of DP1300 recorded at a given *q* = 0.00808 nm^−1^. The experimental data are well represented by single Lorentzian shapes in gradient blue red lines. c) The corresponding effective longitudinal sound velocity *c* as a function of the input laser power. The resulted temperature increase in the light spot is calculated by d*T*/d*P* and denoted by the gradient backgrounds. Inset: transmission electron microscopy (TEM) image of a monolayer of DP1300 illustrating a high degree of structural uniformity. Panels d,e) show the power‐dependent effective longitudinal sound velocity *c* of DP530 and DP2480, respectively. The patterned area in (e) denotes the glass transition. The TEM images of the ordered opals are shown as insets. Scale bars are 200 nm.

To validate the hypothesis that local heating in the films was responsible for the softening, the surface temperature profile of the films (which is considered to be uniform across the film normal due to the small film thickness) was determined using an IR camera (7.5–14 µm, Infratec VarioCAM HD research), equipped with converter lens 0.5×. In this configuration, the minimum spatial resolution was 29 µm at a working distance of 33 mm and the temperature resolution was 0.02 K. The analysis revealed that the surface temperature *T*_S_ increased (in the case of DP1300) by 50 K at the maximum input laser power *P*_max_ = 150 mW (see Figure S4, Supporting Information). This temperature increase is lower than that determined from the BLS‐measured sound velocities, which revealed Δ*T* ≈ 75 K. The discrepancy might be related to BLS collecting the scattered light from the entire scattering volume, whereas the IR camera is sensitive to the light emanating diffusely from the top surface. We note that the Δ*T* for a given film depends on the laser power but not the irradiation time. The film (i.e., DP1300) reaches a thermal equilibrium state within a few seconds as shown in Figure S4c and Video S1, Supporting Information, which is much shorter than the acquisition time of a well‐resolved BLS spectrum (>1 min). This confirms that the films were in thermal equilibrium under the reported conditions and that they experienced no photodegradation even after long exposure to the light.

In the following, we use the BLS results to quantitatively determine the local temperature (*T*_loc_) changes. Using the linear *c*(*T*) relations for the particle brush systems reported in our previous work,^[^ ^16^ ^]^ the *T*_loc_ increase during irradiation with different input powers could be calculated via (d*T*/d*P*) = (d*T*/d*c*)(d*c*/d*P*). This unique way to access the local temperature evolution in situ during irradiation is critical for the following calculation of the absorption coefficient. For example, for DP1300 *T*_loc_ was found to increase from 294 to 369 K (i.e., Δ*T* = 75 K) as the power was increased from 0 to 150 mW (see Figure 1b).

Figure 1d,e displays the power‐dependent sound velocities in DP530 and DP2480, two soft opals with different lattice periods. Specifically, for DP2480 the lattice spacing was determined to be *d* = 275 nm, significantly larger than the value for DP530 (*d* = 163 nm) or DP1300 (*d* = 246 nm). For DP530, the *c* of the longitudinal acoustic mode measured at the same incident angle α = 20° (*q* = 0.00808 nm^−1^) revealed a weak power‐dependence, and the increase of *T*_loc_ (∆*T*) was determined to be about 13 K at *P*_max_ = 150 mW (Figure 1d). Interestingly, whereas a linear trend of *c*(*P*) was observed for DP530 and DP1300, the *c*(*P*) of DP2480 revealed a kink below *P*_max_ (Figure 1e). The increases of d*c*/d*P* but decreases of d*T*/d*P* at *P* ≈ 100 mW could be attributed to the occurrence of glass transition (i.e., *T*_loc_ = *T*_g_) and the associated decrease in mass density at *T* > *T*_g_. The position of the kink was shifted from *P* ≈ 105 mW to *P* ≈ 55 mW as the ambient temperature was increased from 294 to 317 K; however, the corresponding local temperature was found to be constant with *T*_kink_ = *T*_g_ (Figure S5, Supporting Information). Since the sound velocity depends only on temperature (at constant pressure), it was deduced that the position of the kink in *c*(*P*) marks the glass transition. Hence, the BLS results were concluded to be indicative of different levels of optical heating (or light absorption) in the respective brush particle films.

The heating was attributed to radiative energy transfer through optical absorption of the materials. The different (d*T*/d*P*) of the brush particle films pointed to distinct energy transfer efficacies for the distinct material systems. In particular, the high (d*T*/d*P*) of DP2480 indicated a significant increase in the energy transfer (and hence optical absorption). This is an intriguing finding since the concentration of absorbing bromine (Br) end‐groups was the smallest for DP2480. Since all materials were purified using analogous wet chemical procedures to quantitatively remove the residual catalyst (details below), optical absorption was predominantly associated with bromine end‐group functionalities. The latter can be estimated from the composition of the brush particles as *c*_e_ = σ/(*R*/3 + *σ N ν*), with ν = 0.21 ± 0.01 nm^3^ representing the volume of a styrene repeat. Thus, the concentrations of end‐groups for the various material systems were *c*_e_ = 5.23 × 10^−3^ nm^−3^ (DP600), 5.68 × 10^−3^ nm^−3^ (DP530), 3.16 × 10^−3^ nm^−3^ (DP1300), 1.67 × 10^−3^ nm^−3^ (DP2480), 12.42 × 10^−3^ nm^−3^ (DP214), and 3.41 × 10^−3^ nm^−3^ (DP955). To better understand the effects of the structure and composition of the brush particle films on the radiative energy transfer, the optical absorption coefficients of the films were analyzed using FEM simulations.

The different contributions of optical scattering and absorption render a direct comparison of the light absorption performance in our systems difficult. To quantitatively determine the effective absorption coefficient of the films, the radiative power needed to match the measured local temperature rise at the laser spot was determined using FEM simulations. The simulations were based on a 2D‐axisymmetric, steady‐state heat transfer model with a Gaussian, volumetric heat generation profile, and convective/radiative boundary conditions (Figure S6, Supporting Information). The thermal conductivities of the brush particle films were estimated according to the Maxwell model.^[^ ^18^ ^]^ The ambient temperature was set to be 295 K. The thickness of brush films was set to be 100 µm, approximately equal to the experimental systems. The absorption coefficients (ε) were calculated using the Lambert–Beer law, *I*(*z*) = *I*_0_(*z*)*e^*(‐ε*z)^*, where *I*_0_ is the incident heat flux on the top surface and *z* is the distance from the top surface into the sample film. The total absorbed light energy is assumed to be equal to the total volumetric heat generation in the film, which was calculated by integrating *I*(*z*) over the film thickness.

In a first step, the FEM calculation was used to determine the required power of a heat source to recreate the experimentally determined temperature raise, Δ*T*, upon light irradiation at the respective power level of the laser. In a second step, assuming that the photothermal energy transfer is caused by the material optical absorption that obeys the Lambert–Beer law, an effective absorption coefficient of the films was calculated. The model of the ordered structure of the assembled polymer‐tethered particles and the associated temperature profile in the illuminated spot is illustrated in **Figure** **2**a,b. The simulated local temperature *T*_loc_ (= 294 + Δ*T*) in the respective films is shown in Figure 2c. As expected (and confirmed by the experimental data), no temperature rise occurs in films of pristine PS due to the absence of any intrinsic light absorber; the data for pristine PS is thus shown in Figure 2c as a reference. The *T*_loc_(*P*) of the particle brush opals were matched to the experimental temperature value determined from *c*(*P*) and *c*(*T*). The increasing slopes imply increasing energy dissipation through optical absorption. The kink in the sound velocity versus power plot observed in both DP2480 and DP955 represents the glass transition of the opal films. Figure 2d shows the effective absorption coefficients (ε_opal_) of the brush particle films that were determined from the FEM analysis by matching the experimental and simulated *T*_loc_(*P*) of the films. Interestingly, the values of ε_opal_ display an opposite trend to the absorption coefficient ε_Br_ of the individual brush particles. The latter was determined on the basis of the bromine end‐group concentration *c*_e_ given in the preceding section and assuming a molecular absorption coefficient ε_Br_ = 22.5 L mol^−1^ cm^−1^ for an individual bromine moiety.^[^ ^19^ ^]^

**Figure 2:**
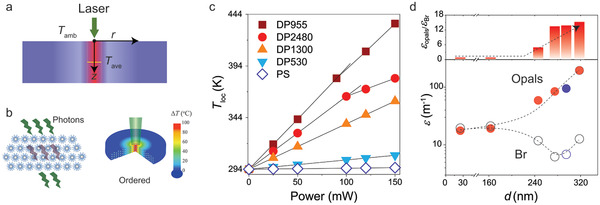
Determination of the structure‐induced effective optical absorption coefficients by finite element method (FEM) simulations. a) Schematic of the 2D model used in the calculations. b) Illustration of the temperature profile in the ordered structure of assembled polymer‐tethered particles (scheme). The temperature increase Δ*T* is indicated by the color scale. c) Calculated local temperature *T*_loc_ (= 294 K + Δ*T*) with the input light power for four particle‐brush opals (solid symbols) with the same thickness of 100 µm. The featured kinks in DP2480 and DP955 corresponding to the glass–rubber crossover are guided by lines. The light absorption enhancement effect is absent in the PS film (open symbols). d) Calculated absorption coefficient ε of the opals and the intrinsic Br absorber as a function of the intercore distance. The corresponding absorption coefficient ratio ε_opals_/ε_Br_ is shown in the upper panel. The blue circle refers to the DP1300 film plasticized with 20 wt% dimethyl phthalate. The dashed lines are guides to the eye.

Different origins for optical absorption were considered. One potential source for the optical absorption in materials synthesized by SI‐ATRP is the residual copper catalyst. To prevent significant amounts of catalyst, all materials were purified by dilution of the reaction solutions with 10 times tetrahydrofuran, subsequently passing of solutions through 1:1 vol neutralized Al_2_O_3_ columns and finally precipitation in cold methanol. The ultraviolet–visible (UV‐vis) spectra of the brush particle solutions (Figure S7, Supporting Information) did not reveal detectable amounts of the residual copper (Cu) catalyst, which implies that this component did not make a sizable contribution to optical absorption. We note that chemical purification processes—such as those used in our work—can never be absolutely quantitative. However, while we cannot exclude the presence of minute amounts of Cu catalyst, this result clearly suggests that a potential contribution of residual Cu‐catalyst to light absorption is negligible compared to the contribution of bromine end group functionalities. The calculated absorption coefficient ε_opal_, which was determined from FEM analysis for films with spacing *d* < 200 nm, is in good agreement with the expected absorption of “regular solutions” of bromine moieties. This implies that the total absorption of (non‐photonic) brush particle films with spacing *d* < 200 nm is well described by assuming a “regular solution” of absorbing moieties (Figure 2d). To examine the reproducibility of the observed effect, DP1300 is swollen through plasticization by dimethyl phthalate with negligible optical absorption at λ = 532 nm. The increase of the spacing (inter‐core distance) leads to a redshift of the photonic stop compared to the pristine DP1300 case and hence toward the laser wavelength (532 nm). The absorption coefficient of the swollen DP1300 (blue symbol in Figure 2d) is expectedly enhanced, which thereby emphasizes the robustness of the observed light–matter effect.

With increasing the periodicity, a significant increase of ε_opal_ was observed despite the decrease of ε_Br_ as a consequence of the smaller *c*_e_. The absorption enhancement is quantified by the amplification factor ε_opal_/ε_Br_. As revealed in the upper panel in Figure 2d, it assumes a maximum of 16 for DP955. For the systems featuring enhanced absorption, we found a similarity in their UV–vis spectra, that is, the edge of the film photonic stopband was close to the laser wavelength λ = 532 nm (inset of Figure 4b). For instance, the extinction spectra of DP955 (black squares in Figure 3b) revealed a (weak) stopband at λ ≈ 600 nm; the upper (blue) band edge was located at about λ ≈ 540 nm. Since at the band edge the group velocity of the light is reduced, this observation suggested that the absorption enhancement is due to a slow‐photon effect.^[^ ^10a^ ^]^ The prolonged residence time of light raises the probability of photon absorption and thus the energy transfer efficacy in brush particle films assembled from DP955. We note that the enhancement effect was absent in the systems without a stopband (such as DP600 or pure PS) or in systems with a larger distance between the edge of the stopband and the laser wavelength (e.g., DP530).

**Figure 3:**
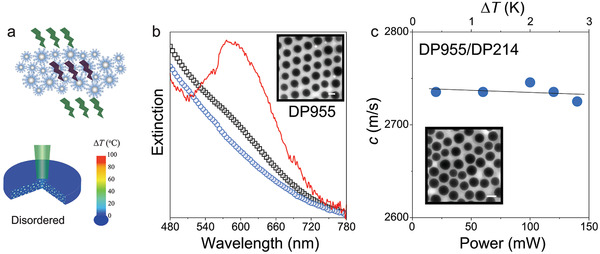
Absence of optical absorption in disordered films of mixed brush particles. a) Scheme of the disordered structures of a particle brush blend (DP955/DP214) and absence of heating effect (Δ*T* = 3K). b) UV–vis extinction spectra of DP955 (black) and the mixed binary DP955/DP214 (blue symbols) assemblies. The red line is the spectrum of DP955 after correcting the scattering contribution. Inset: TEM image of a monolayer of the ordered DP955 film. c) Power‐independent sound velocity in the binary DP955/DP214 mixture film. Inset: TEM image of a monolayer of the disordered DP955/DP214 film. Scale bars are 200 nm.

To confirm the slow‐photon effect, we examined the power‐dependent temperature profile in films formed by a 50 wt%/50 wt% binary mixture of DP955/DP214 schematically shown in **Figure** **3**a. The absence of long‐range order in the disordered DP955/DP214 film was confirmed by TEM (inset of Figure 3c). Accordingly, no indication of stopband formation was observed by spectrophotometry (blue circles in Figure 3c). The sound velocity (black squares in Figure 3c) in the DP955/DP214 blend system was found to be almost constant with the increasing power, suggesting that irradiation‐induced heating was negligible (Figure 3c, schematically shown in Figure 3a). This is a pertinent finding since both components, in their pure state, displayed significant absorption enhancement. It was thus concluded that the microstructure of the films was essential to afford the photothermal energy transfer. An alternate mechanism, that is, enhanced absorption due to multiple scattering and the associated increase in the path length, could be ruled out by comparing the distinct brush particle systems.^[^ ^20^ ^]^ Specifically, the higher number density of the particle cores (i.e., scattering centers) and chain ends (i.e., absorption centers) should result in a more pronounced amplification effect for DP955/DP214. However, this trend was inconsistent with the experimental trend shown in Figure 3a,c. Thus, it was concluded that the absorption enhancement is a consequence of the slow photon effect that is correlated with the photonic properties of the brush particle films. This finding was surprising, since the rather small refractive index contrast in the silica/PS hybrid systems only allows for a modest width of the stopband as seen in Figure 3b. To the best of our knowledge, the significant enhancement (by a factor of ≈16) in the particle brush opals presents the highest reported amplification related to the slow‐photon effect for self‐assembled systems.

To further support the proposed slow‐photon mechanism of the observed absorption enhancement, the dependence of the temperature increase on the angle‐of‐incidence was evaluated. All systems exhibiting a stopband in the visible range revealed a blue shift in the position of the stopband with increasing the incident angle. The associated shift of the band edge position is expected to alter the absorption enhancement. To determine the effect of the stopband position on the slow‐photon effect, measurements of *c*(*P*) were performed at different angles of incidence as shown in **Figure** **4** and Figure S8, Supporting Information. Figure 4a shows the power‐dependent *c* of DP955 (the system with the highest amplification of absorption) at three incident angles. Figure 4a reveals a shift of the “kink” of *c*(*P*) toward higher powers as the angle of incidence was increased. This demonstrated the decrease of the amplification factor with the increasing angle of incidence, strong support for the “photonic” origin of the absorption enhancement in the brush particle films.

**Figure 4:**
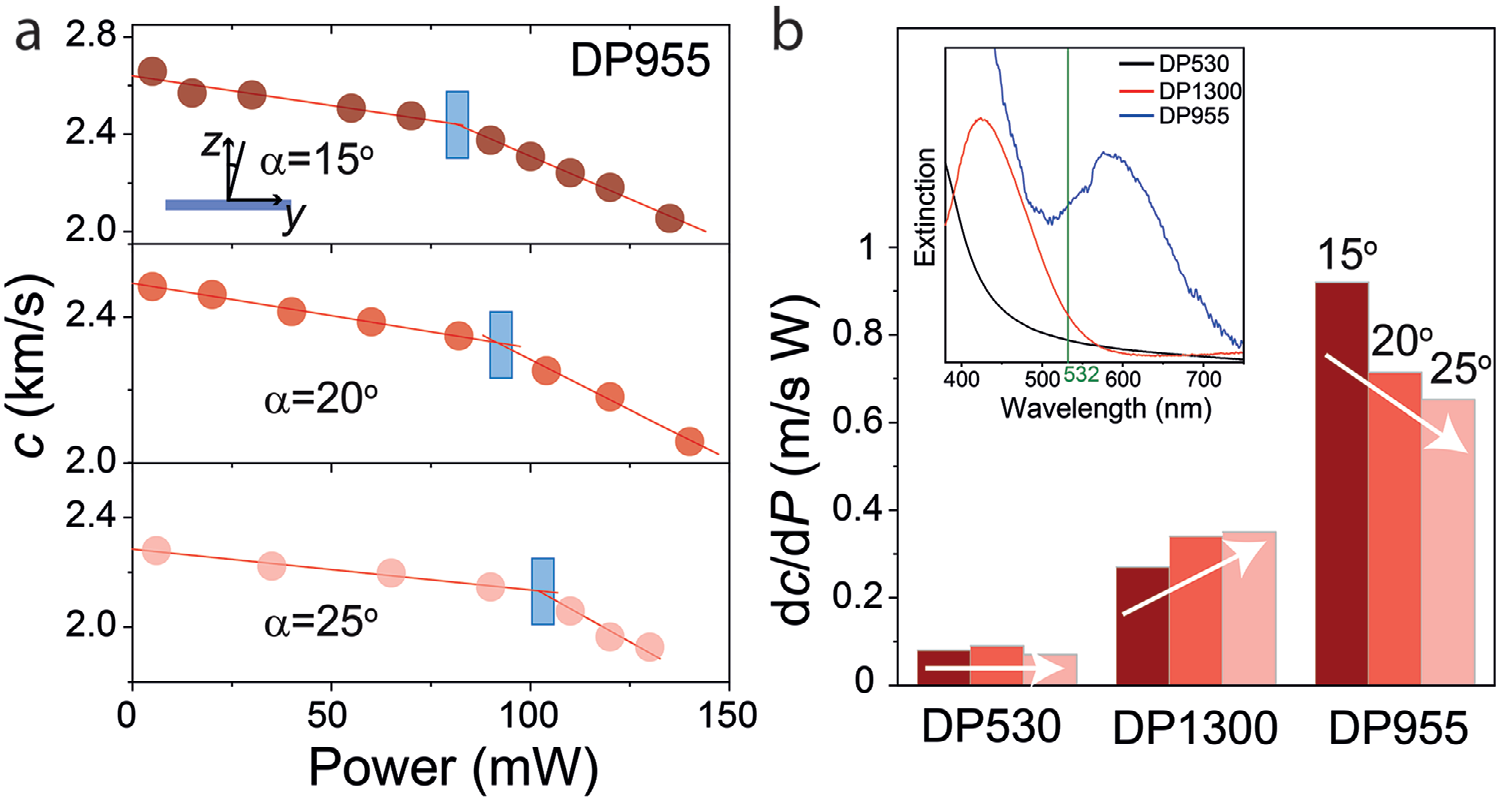
Slow photon facilitated light absorption in polymer‐tethered colloids. a) The dependence of the longitudinal sound velocity *c*(*P*) in DP955 on the laser light power at three incident angles. The threshold power for the glass–rubbery transition (indicated by the shaded areas) increases with the angle of incidence. Inset: Illustration of the definition of the incident angle α. b) The rate of the decrease of the sound velocity with the light power for the DP530, DP1300, and DP955 opals at three incident angles (15°: dark red, 20°: red, 25°: light red). The trend indicated by the arrows is inverted from DP1300 to DP955. Inset: UV–vis spectra of DP530, DP1300, and DP955 (after subtracting the contribution of particle scattering). The green line in the inset represents the laser wavelength λ = 532 nm.

The conclusion of the slow‐photon origin of the observed absorption enhancement is further supported by the analysis of the dependence of d*c*/d*P* on the angle of incidence (note that a larger value of d*c*/d*P* indicates a stronger absorption in the material). Two scenarios for d*c*/d*P* as a function of the angle of incidence, α, can be expected. In uniform (non‐photonic) materials with absorption, d*c*/d*P* is expected to increase with the angle of incidence due to the increased path length of the light within the film. This contribution is considered to be insignificant in the present case because the examined limited range of incident angles merely results in a maximum variation of the path length by about 60%. To explain the strong effect of the angle variation on the light adsorption in photonic materials, a more substantial contribution is expected to be the variation of light–matter interactions in materials with different stopband positions. Figure 4b summarizes d*c*/d*P* at three incident angles (15°, 20°, and 25°) for three particle‐brush systems (DP530, DP1300, and DP955) in the glassy state. The brush systems represent three limiting cases, that is, no stopband near λ = 532 nm (DP530) and matching of the lasing wavelength with the lower (DP1300) and upper (DP955) stopband edges, respectively. Interestingly, Figure 4b reveals striking differences in the trend of d*c*/d*P* depending on the stopband position. In the case of DP530, no definite trend in d*c*/d*P* could be detected. This confirms that the increased path length does not contribute significantly to the photothermal energy transfer. In contrast, the absorption was observed to significantly increase (≈38%) in the case of DP1300, while in the case of DP955 a pronounced decrease (≈41%) was observed as the angle α was increased from 15° to 25°. Since the transmitted power of the vertically‐polarized incident light merely decreases by 0.8% as α increases from 15° to 25° (1.2 mW for *P*_input_ = 150 mW), we could infer that the variation of the absorption mainly results from the angle‐dependent slow photon effect. To rationalize the opposing trends for DP955 and DP1300, we note that increasing α is equivalent to employing a probing laser with a longer wavelength. Thus, for DP1300, increasing α corresponded to a shift toward the lower band edge, which is consistent with the enhancement of absorption due to the slow photon effect. In contrast, the shift toward the stopband center in the case of DP955 leads to increased reflection and thus a reduction in the light absorption. We noted that varying the angle of incidence is not only a practical way to change **q** but also serves as a “reference experiment” to confirm that the reported effect is not caused by intrinsic absorption. Since the light path length increases with increasing the deviation from the film normal, one would expect to see an increase in Δ*T*—this is in contrast to our “photonic system” that featured a decrease. The angle‐dependent absorption characteristics revealed in Figure 4b strongly support the proposed absorption enhancement mechanism by slow photon coupling.

The details of the light–matter interactions of hybrid brush particles remain a subject of current study. Our results, specifically, the angle dependence of the heating effect, clearly point to the “photonic” origin of the observed energy transfer. According to the common understanding of photonic crystal structures, the group velocity of light experiences slow‐down at frequencies near the edge of the bandgap of a photonic crystal. The increased residence time of light increases the probability of absorption and thus the photothermal energy conversion. We hypothesize that this effect is also fundamental to our reported results. However, what is surprising is the strength of the effect which exceeds previous reports on other types of photonic crystals which required the addition of dyes to achieve comparable photothermal energy conversion.^[^ ^7^, ^21^ ^]^ We hypothesize that this difference is related to the microstructure of brush particles which differs from those of the previously studied systems. However, further elucidation of this aspect will require supplementary theoretical investigations.^[^ ^17^ ^]^ Since the slow‐photon effect in photonic crystal structures is sensitive to both the dielectric contrast and characteristic length scales, we envision that our approach could be extended to other wavelengths by suitable engineering of the brush particle building blocks.

Our results revealed a pronounced photothermal energy transfer process in self‐assembled brush particle films. The sensitive dependence of the energy‐transfer process on the microstructural details (i.e., length scales and degree of order) of the film, the angle of incidence and the photonic stopband position identifies the heating effect as being related to the slowdown of the photons. Heating occurs if the wavelength of the incident light aligns with the band edge of the film. Interestingly, an amplification factor of 16 is observed for a (suitably designed) brush particle film. This value exceeds previously reported slow‐photon‐induced absorption enhancement in self‐assembled colloidal materials by about an order of magnitude and rationalizes why rapid softening of the films is observed even for materials with an infinitesimal amount of intrinsic absorbers. The high absorption enhancement in brush particle films is interesting from a fundamental and applied perspective. First, similar amplification factors have been observed previously only for micro‐engineered highly dielectric or metallic photonic crystal structures with nearly complete stopbands. The origin in brush particle films with a significantly smaller dielectric contrast and a lower degree of order is unclear. Interestingly, similar effects were not observed in regular core‐shell opal films consisting of silica colloids embedded within a polymer matrix (Figure S9, Supporting Information).^[^ ^22^ ^]^ This raises the possibility that the observed slow photon effect is related to the altered chain conformations in brush particle systems, specifically the more stretched conformations near the particle surface and its effect on the light–matter interactions. For example, it was recently demonstrated that densely tethered brush layers give rise to novel phononic bandgap characteristics in brush particle films that are not observed in regular colloidal assembly structures.^[^ ^17^ ^]^ Since the concept in this work is not expected to depend on the core composition and hence should be applicable to a wide range of material compositions, we envision that the reported results will spark future research in areas such as “optics‐driven” microfabrication of polymer hybrid materials. Note that many additive manufacturing methods depend on localized heating of materials; extending these processes to optically‐modulated heating could benefit feature resolution. This points to novel opportunities for controlling light–matter interactions in polymer hybrid materials.

## Experimental Section

The Experimental Section is included in Supporting Information.

## Conflict of Interest

The authors declare no conflict of interest.

## Supporting information

Supporting Information

Supporting Video 1
