# Supplementary material for: Transparent Hybrid Opals with Unexpected Strong Resonance‐Enhanced Photothermal Energy Conversion
Source: Adv Mater. 2020 Nov 30;33(2):2004732. doi: 10.1002/adma.202004732 (PMC11468544; doi:10.1002/adma.202004732)
Supplement: Supplementary file 1 — Supporting Information [file ADMA-33-2004732-s001.pdf]

© 2020 Wiley-VCH GmbH

ADVANCED MATERIALS

Supporting Information

for *Adv. Mater.*, DOI: 10.1002/adma.202004732

Transparent Hybrid Opals with Unexpected Strong Resonance-Enhanced Photothermal Energy Conversion

Yu Cang, Jaejun Lee, Zuyuan Wang, Jiajun Yan, Krzysztof Matyjaszewski, Michael R. Bockstaller, and George Fytas**

© 2020 Wiley-VCH GmbH

Supporting Information

Transparent Hybrid Opals with Unexpected Strong Resonance-Enhanced Photothermal Energy Conversion

Yu Cang, Jaejun Lee, Zuyuan Wang, Jiajun Yan, Krzysztof Matyjaszewski, Michael R. Bockstaller, George Fytas**

Materials and Methods

Synthesis of particle brush: Particle brush synthesis was performed using surface-initiated atom transfer radical polymerization following a procedure described previously.[1] The final molar ratios of reaction components in a typical reaction were approximately $[Styrene]_0:[SiO_2\text{-}Br]_0:[CuBr]_0:[CuBr_2]_0:[PMDETA]_0$ of 2000:1:2.5:0.25:2.75 with a volume fraction of non-reactive solvents of 5.4% dimethylformamide and 40% anisole in a 100 mL flask and stirred at approximately 1000 rpm. The polymerization was stopped by exposing the catalyst to oxygenated tetrahydrofuran after cooling under continuous stirring. The final product was dialyzed against tetrahydrofuran and methanol until the copper (II) catalyst was removed as evidenced by disappearance of its characteristic color.

Styrene (St, Aldrich, 99%) was purified by passing through a basic alumina column before use. Copper (I) bromide was prepared by reduction of an aqueous solution of $CuBr_2$ with an aqueous solution of ascorbic acid, while copper (I) chloride was prepared by reduction of $CuCl_2$ aqueous solution using an aqueous solution of sodium sulfite. Both copper (I) halides were then sequentially filtered, washed with methanol, dried and stored under vacuum before use. Silica nanoparticles (SiO_2NP), 30% solution in isopropanol, effective diameter, $d_{TEM} \approx 113$ nm, were donated by Nissan Chemical Corporation and used as received. 5-hexen-1-ol (98%), α-bromoisobutyryl bromide (98%), triethoxysilane (95%), ethyl 2-bromoisobutyrate (EBiB, 98%), 4,4′-dinonyl-2,2′-bipyridine (dNbpy, 99%), *N*,*N*,*N*′,*N*″,*N*″-pentamethyldiethylenetriamine (PMDETA, 99%), and anisole (99%) were purchased from Aldrich and used as received. All other chemicals and solvents were supplied by Aldrich and Acros Organics.

Fabrication of core-shell opals: These opals were prepared out of the hard core (silica) / soft (poly(ethyl acrylate) (PEA)) shell particles using a melt compression method.[2] The silica (SiO_2)

cores formed an fcc lattice, and the PEA shell served as a homogeneous matrix. The intercore distance $d = (0.74)^{1/3}d_{\mathrm{CSP}}$ of the opals depends on the diameter of the core-shell particles, $d$csp. The core-shell particles consist of a silica core with a diameter of 216 nm and d_{csp} is chosen to be between 252 and 450 nm.[3] The preparation began with the coagulation of the latex dispersion in a rubbery mass. Since PEA is an elastomer with a low T_g, upon heating the shells formed a continuous matrix, in which the silica cores were dispersed.[2]

Size exclusion chromatography (SEC): The molecular weight and dispersity were measured by size exclusion chromatography using a Waters 515 pump and Waters 2414 differential refractometer and PSS columns (Styrogel 10^5, 10^3, and 10^2 Å) in THF as an eluent (35 ^{o}C, flow rate of 1 mL/min) with toluene and diphenyl ether as internal references. A linear polystyrene (PS) standard was used for calibration. To perform SEC, chains were cleaved from particles by etching of particles in HF in a polypropylene vial for 20 h, neutralized with ammonium hydroxide, and dried with magnesium sulfate before running SEC. Hydrofluoric acid (50 vol% HF) was purchased from Acros Organics and used as received. THF was purchased from Aldrich and used as received.

Brillouin light spectroscopy (BLS): BLS detects the scattering of an incident probing beam from the thermally activated density fluctuations (phonons) along $\mathbf{q} = \mathbf{k_i} - \mathbf{k_s}$ determined by the scattering geometry, where $\mathbf{k_i}$ and $\mathbf{k_s}$ are incident and scattered photon wave vectors respectively. The scattering wave vector $\mathbf{q}$ couples to the thermal phonons along the same direction with an equal wave vector. The associated phonon energy is represented by the frequency shift $\pm f$ of the inelastically scattered light resolved by an actively stabilized tandem FP interferometer (JRS Instruments). The sound velocity can be calculated as $c = 2\pi f/q$. By tuning the polarization of the probing light (V or H), the polarization of acoustic phonons at a given direction could be selected,

where V (H) denotes the polarization vertical (horizontal) to the scattering plane defined by incident and scattered light wave vectors. In this work, we employed the transmission scattering geometry (schematically shown in Figure 1) to resolve the phonon propagation in the isotropic film, where $q = \frac{4\pi}{\lambda}\sin(\alpha)$ is independent of the refractive index with α being the incident angle. We combined V-polarized incident and scattered light to select longitudinal phonons. We note that the high optical stability of the BLS over weeks warrants an absolute experimental error less than 2% and much lower relative error in the sound velocity**.** Concurrently, the fast kinetics of the effect along with the ultra-stability of the opals act synergistically in a reproducible experiment in different spots of the samples and different repetitions in the same spot.

Finite element method (FEM) simulations: FEM simulations were conducted to determine the absorption coefficients of the soft opal films. Because the films have different thicknesses, knowing the absorption coefficients further allows comparing the temperature increases in the laser-spot region under the same film thickness. The simulations were based on a 2D-axisymmetric, steady-state heat transfer model with a Gaussian, volumetric heat generation profile and convective and radiative boundary conditions (Figure S6a). The film radius (R_2) was assumed to be 2 mm. The film thickness (H) was set to be the measured film thicknesses. The densities and specific heats of the films were calculated using a volume-fraction-based averaging relation. The thermal conductivities were calculated according to the Maxwell model as $k_{eff} = k_{PS}\frac{(k_{SiO2}/k_{PS}+2)+2(k_{SiO2}/k_{PS}-1)\phi_{SiO2}}{(k_{SiO2}/k_{PS}+2)-(k_{SiO2}/k_{PS}-1)\phi_{SiO2}}$, where ϕ_{SiO2} is the vol% of SiO_2, k_{SiO2} = 1.4 W m^{-1} K^{-1}, and k_{PS} = 0.15 W m^{-1} K^{-1}. The Gaussian profile reads $\dot{q}(r,z) = \left(\frac{2\varepsilon P}{\pi R_1^2}\right)\exp\left(-\frac{2r^2}{R_1^2}\right)\exp(-\alpha z)$ [W/m^3], where ε is the absorption coefficient (m^{-1}), P is the laser heating power (W), and R_1 is the

radius of the laser spot. For the convective boundary conditions, heat transfer coefficients for horizontal and vertical walls were used. For the radiative boundary conditions, all surfaces were assumed to be diffuse and have an emissivity of one. The ambient temperature (T_∞) was set to be 295 K. The average temperature in the disk, illustrated by the yellow segment in Figure S6a, was considered as the average temperature (T_{loc}) in the laser spot and compared with the experimental values. The absorption coefficient α was varied to achieve an agreement between the experimental and simulated T_{ave} within 0.1 K. The absorption coefficients of all soft opal films were determined and subsequently used to calculate the T_{ave} at a unified film thickness of 100 μm. Figure S6b shows the calculated dT_{ave}/dP vs. film thickness for eight soft opals.

Table S1. Elastic moduli (GPa), Poisson's ratio and thickness (µm) of PS-grafted SiO_2 particle brush systems in this work.

Sample ID*	Shear modulus	Longitudinal modulus	Bulk modulus	Young's modulus	Poisson's ratio	Thickness of film
DP600	3.11	9.20	5.05	7.57	0.25	46
DP530	3.32	11.79	7.36	8.67	0.30	45
DP1300	1.67	6.53	4.30	4.44	0.33	100
DP2480	1.59	6.26	4.14	4.24	0.33	105
DP955	2.18	8.58	5.67	6.13	0.32	40
DP955/DP214	2.41	9.48	6.26	6.77	0.31	70

*The microstructure of brush particle films was discussed as part of previous publications.[4]

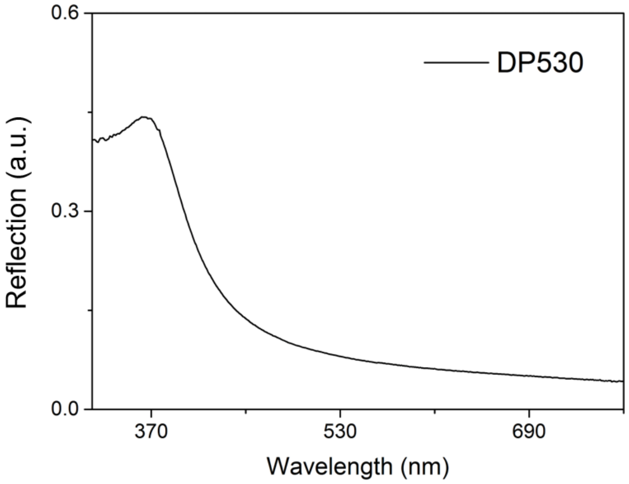

Figure S1. Reflection spectra of DP530.

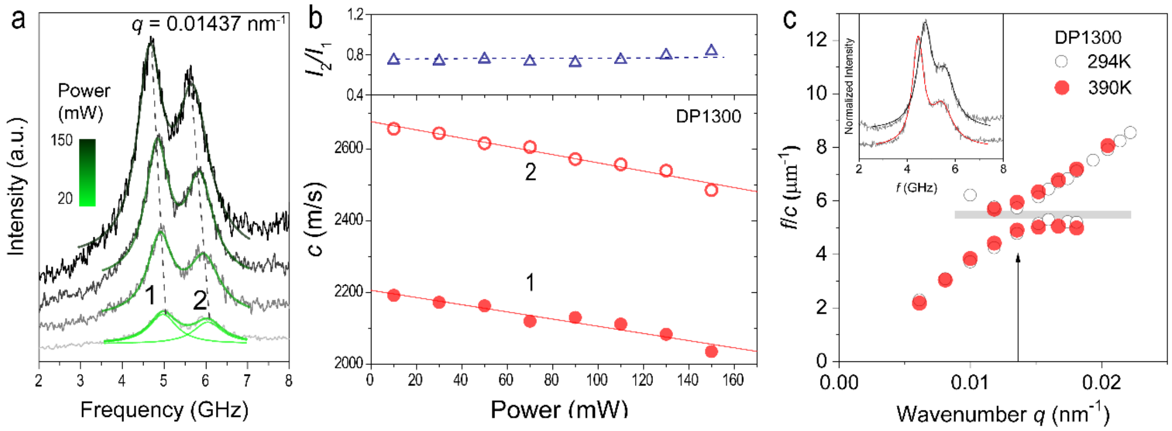

Figure S2. (a) Evolution of the power-dependent BLS spectra (black lines) of DP1300 recorded at q = 0.01437 nm^{-1}. The observed two modes are denoted as 1 and 2, which are well represented by Lorentzian fits in gradient green lines. (b) The corresponding sound velocity $c = 2\pi f/q$ of mode 1 and 2 as a function of the input power. The intensity ratio I_2/I_1 of mode 1 and 2 is independent of the input power as shown in the upper panel of (b). The dashed lines are guide for the eye; the solid lines represent linear fits of $c(P)$. (c) Normalized dispersion relation, $f/c(q)$, of DP1300 recorded at T=294 and 390K respectively. Inset: Normalized BLS spectra at the q indicated by arrow recording at T=294K (black Lorentzian fitting) and T=390K (red Lorentzian fitting). The shaded area denotes the phononic bandgap.

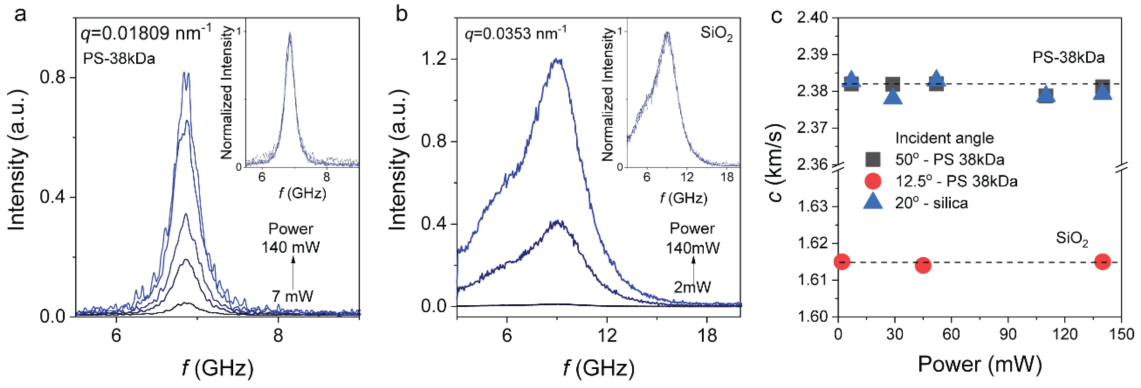

Figure S3. BLS spectra of the PS-38kDa film at q = 0.01809 nm^{-1} (a) and the silica film at q = 0.0353 nm^{-1} obtained at selected powers of the probing laser. Inset: the normalized spectra at selected powers completely overlap suggesting their power-independent features. Note that the measurements on the silica films were performed at the same incident angle but in the backscattering geometry, in which $q = 4\pi n/\lambda$ with n = 1.32. (c) Power-independent longitudinal sound velocity $c = \frac{2\pi f}{q}$ in the PS-38kDa and silica films, where f is extracted from the Lorentzian fits in (a) and (b). For PS-38kDa, the measurements were performed at two incident angles to confirm the angle independence of non-absorbable films. The horizontal dashed lines are guides to the eye.

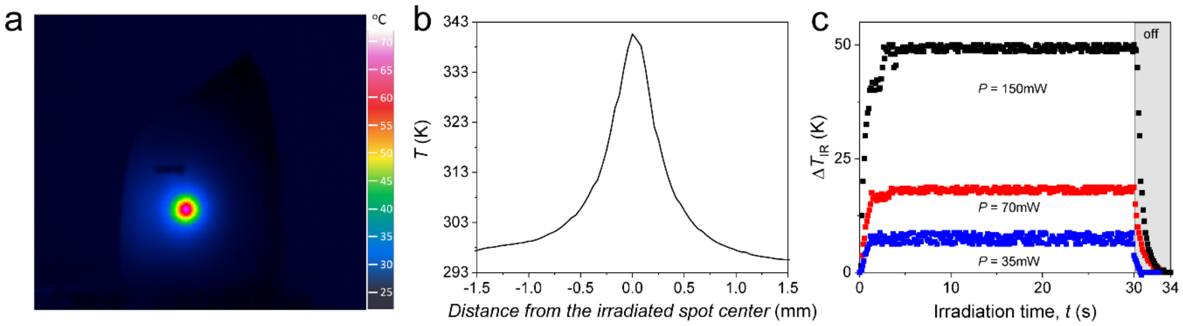

Figure S4. (a) Thermal imaging of laser spot in DP1300 at P = 150 mW using an infrared camera. (b) Temperature profile through the center of the laser spot across the top surface of the film. (c) The temperature increase, ΔT at the spot center, obtained from Infra-Red (IR) thermal imaging in Figure S4a and b, as a function of irradiation time at different input power P. The shaded area denotes temperature decaying as the light is off.

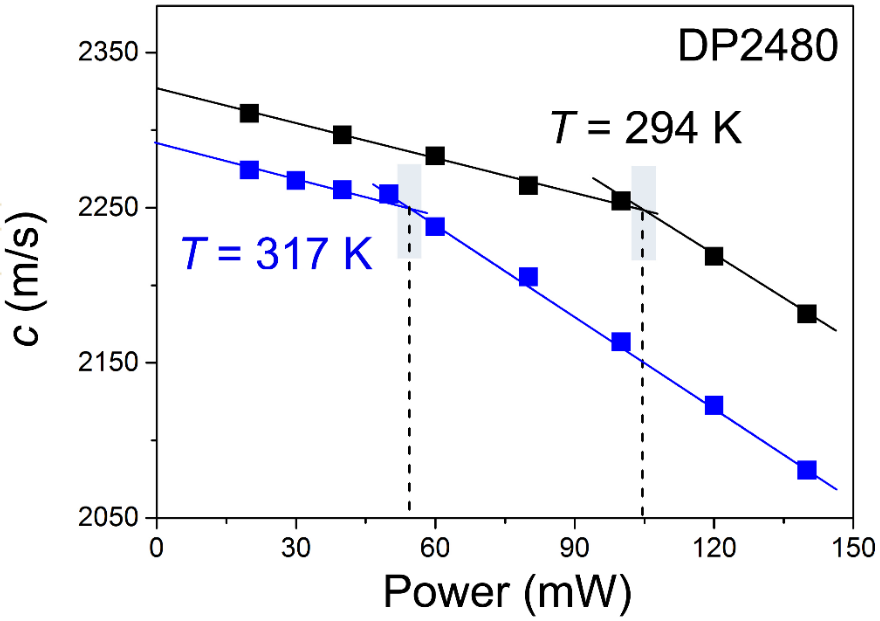

Figure S5. Longitudinal sound velocity *c* of DP2480 *vs.* input power at environmental temperatures of 293 and 316 K, respectively. The shaded areas denote the glass transition occurring at the powers indicated by the dashed lines. The solid lines represent linear fits of $c(P)$ below and above glass transition.

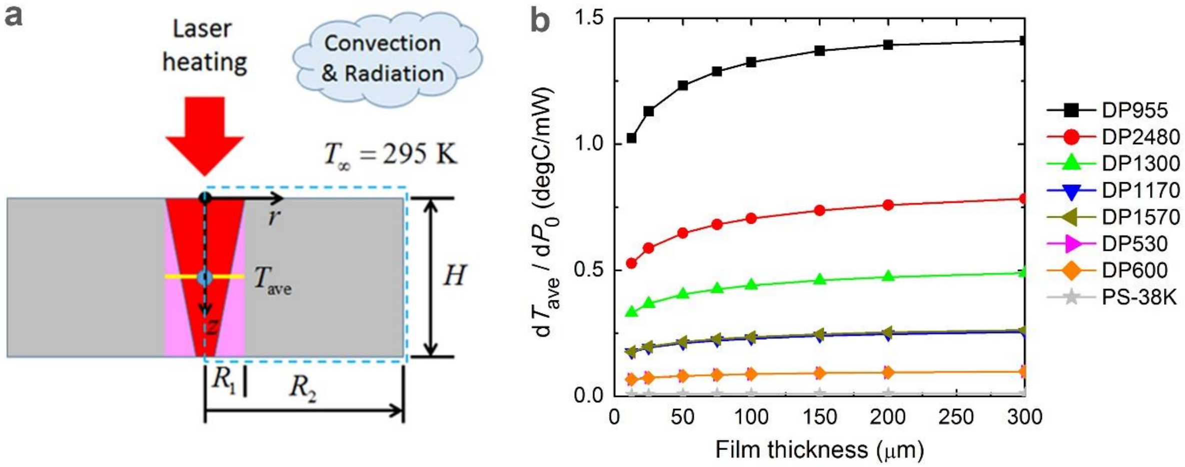

Figure S6. Finite element method (FEM) simulations. (a) Schematic of the simulation domain. The simulations were based on a 2D-axisymmetric, steady-state heat transfer model with a Gaussian, volumetric heat generation profile and convective and radiative boundary conditions. See the text for more information. (b) Calculated dT_{ave}/dP_0 *vs*. film thickness for eight soft opals.

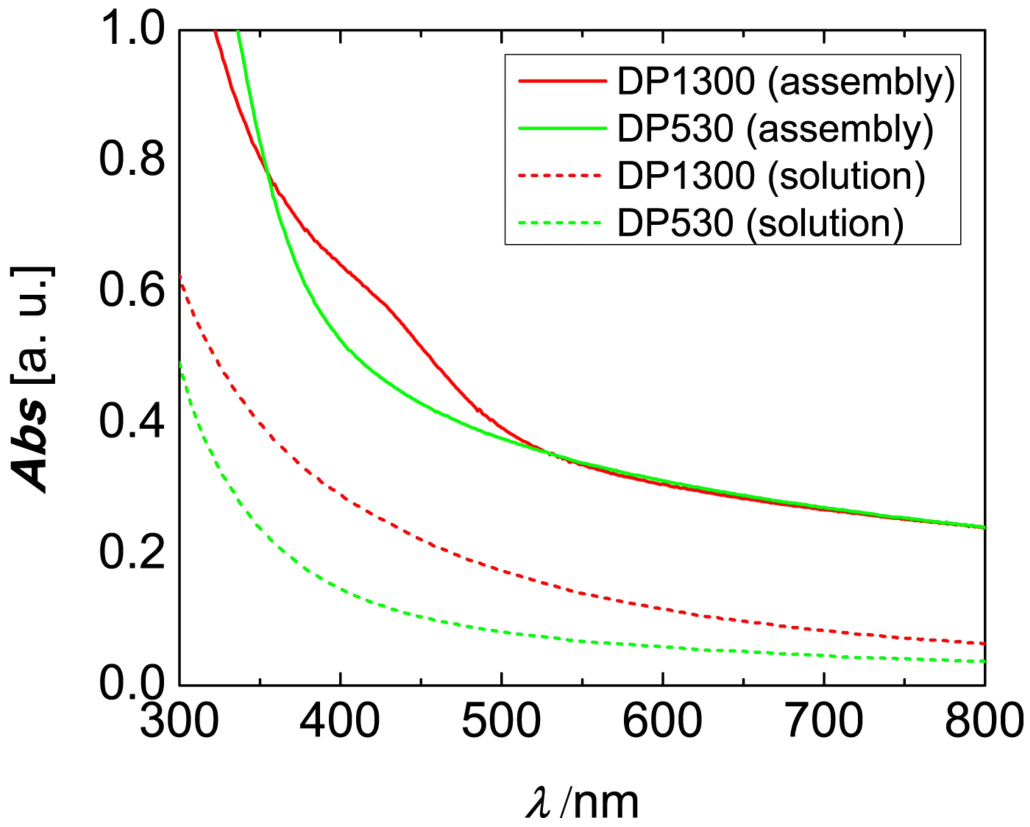

Figure S7. UV-vis spectra of brush particles (DP1300 and DP530) in the assemblies and solution, respectively.

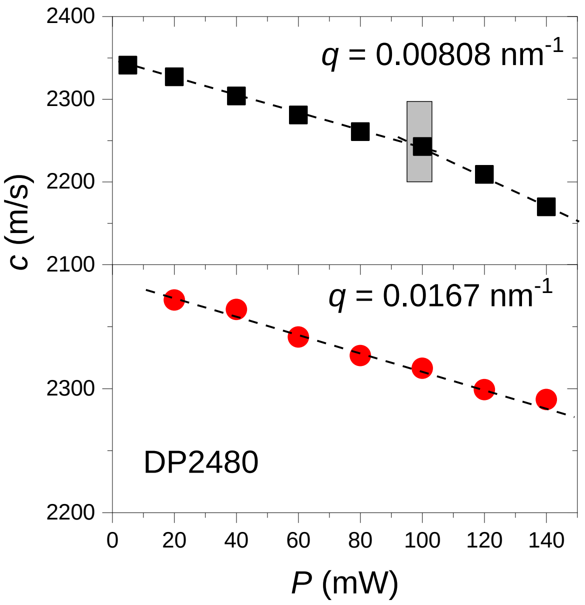

Figure S8. Longitudinal sound velocity *c* as a function of the input power *P* for DP2480 at q = 0.00808 and 0.0167 nm^{-1}, respectively. The shaded area in the upper panel denotes the presence of a glass transition process. The dashed lines are guides to the eye.

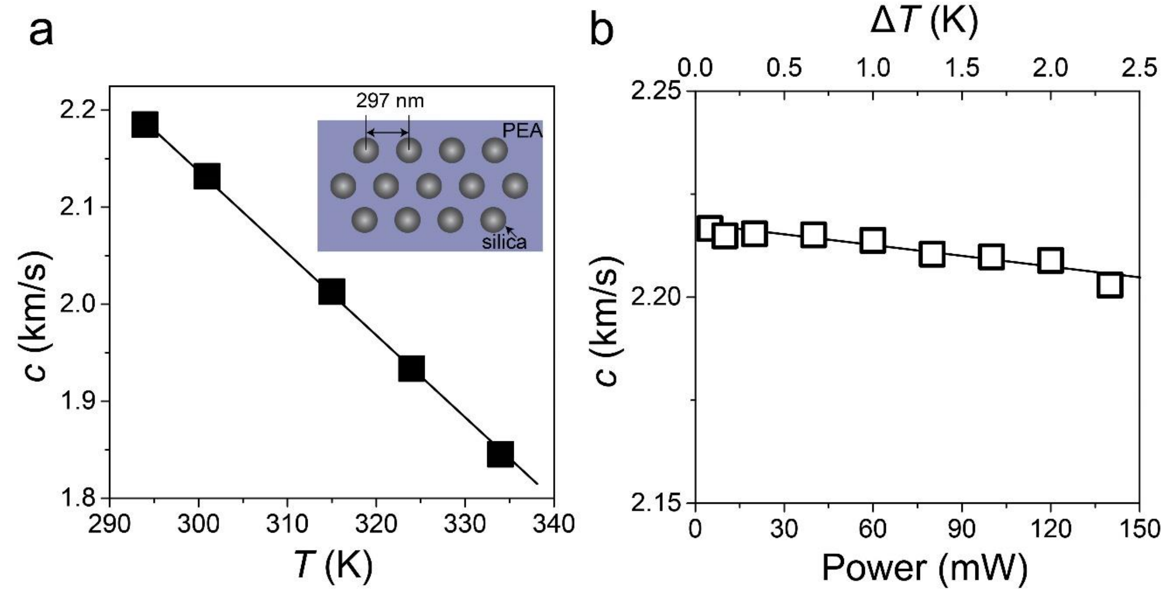

Figure S9. (a) Temperature dependence of longitudinal acoustic sound velocity *c* of the opal consisting of silica particles in a homogenous poly(ethyl acrylate) (PEA) matrix. The silica cores have the fcc ordering with an intercore distance d = 297 nm, which is schematically shown in the inset. The $c(T)$ is well-fitted by the solid, linear line. (b) The power dependence of the longitudinal acoustic sound velocity. The resulting temperature increase in the laser spot is calculated by dT/dP and shown on the top-axis.

References

[1] C. M. Hui, J. Pietrasik, M. Schmitt, C. Mahoney, J. Choi, M. R. Bockstaller, K. Matyjaszewski, *Chem. Mater*. **2014**, *26*, 745.

[2] T. Ruhl, P. Spahn, G. P. Hellmann, *Polymer* **2003**, *44*, 7625.

[3] T. Still, *High frequency acoustics in colloid-based meso-and nanostructures by spontaneous Brillouin light scattering*, Springer Science & Business Media, 2010.

[4] a) J. Midya, Y. Cang, S. A. Egorov, K. Matyjaszewski, M. R. Bockstaller, A. Nikoubashman, G. Fytas, *Nano Lett.* **2019**, *19*, 2715; b) E. Alonso-Redondo, M. Schmitt, Z. Urbach, C. Hui, R. Sainidou, P. Rembert, K. Matyjaszewski, M. Bockstaller, G. Fytas, *Nat. Commun.***2015**, *6*, 1.
